# DNA Methylation Biomarkers in Stool Samples: Enhancing Colorectal Cancer Screening Strategies

**DOI:** 10.3389/or.2024.1408529

**Published:** 2024-07-23

**Authors:** Floriana Porcaro, Serena Voccola, Gaetano Cardinale, Piercarmine Porcaro, Pasquale Vito

**Affiliations:** ^1^ Centro Delta srl, Apollosa, Italy; ^2^ Consorzio Sannio Tech, Apollosa, Italy; ^3^ Tecno Bios srl, Apollosa, Italy; ^4^ Department of Science and Technology, University of Sannio, Benevento, Italy

**Keywords:** colorectal cancer (CRC), DNA methylation biomarkers, stool-based tests, early detection, epigenetics, non-invasive, precision medicine

## Abstract

Colorectal cancer (CRC) is a significant global health challenge, ranking among the leading causes of cancer-related mortality worldwide. Despite efforts in prevention and early detection, CRC incidence and mortality rates are expected to rise substantially. Traditional screening methods like gFOBT, FIT, flexible sigmoidoscopy, colonoscopy, CTC, and colon capsule have limitations, including false positives/negatives, limited scope, or invasiveness. Recent developments in CRC screening involve DNA methylation biomarkers, showing promise in detecting early-stage CRC and precancerous lesions. Stool-based DNA testing is emerging as a noninvasive and convenient method for detecting CRC-associated DNA methylation alterations, offering potential for earlier detection compared to traditional methods. Several commercial stool-based DNA methylation tests targeting different genes associated with CRC have demonstrated varying sensitivity and specificity, some surpassing traditional screening methods. Challenges remain in optimizing their performance and accessibility. This review discusses how DNA methylation biomarkers could enhance CRC screening, and stool-based DNA methylation tests could revolutionize CRC screening practices, comparing them to the gold standard.

## Introduction

### Contextualizing CRC as One of the Leading Causes of Mortality Worldwide

Cancer stands out as a major contributor to mortality, presenting a significant hurdle in the pursuit of extending life expectancy. Lung cancer remained the leading cause of cancer death, with an estimated 1.8 million deaths (18%), followed by colorectal (9.4%), liver (8.3%), stomach (7.7%), and female breast (6.9%) cancers [1]. Colorectal cancer (CRC) is a globally prevalent malignancy, with its occurrence being notably high. However, a substantial portion of CRC cases can be averted by implementing alterations in modifiable risk factors and actively addressing the identification and elimination of precancerous lesions. In the year 2040, it is anticipated that the incidence of colorectal cancer will surge, reaching an estimated 3.2 million new cases and resulting in 1.6 million fatalities [2]. The transition from normal colonic epithelium to a precancerous lesion and eventually to invasive carcinoma necessitates the gradual accumulation of genetic mutations, which can be either acquired (somatic) or inherited (germline), over a span of roughly 10–15 years [3]. This time frame provides us with the opportunity to conduct screenings and identify both adenomatous polyps (pre-neoplastic, i.e., adenomatous polyps or sessile serrated lesions [4]) and colorectal cancer at an early stage [5]. Therefore, screening tests play a crucial role in colorectal cancer as they can help identify precancerous lesions, can significantly improve both survival rates and quality of life. In contrast to the 14% 5-year survival rate observed in patients with CRC in distant stages, those diagnosed with early-stage CRC exhibit a more favorable prognosis [6]. Symptoms of colorectal cancer typically emerge in advanced stages, indicating significant disease progression. These may include changes in bowel habits, rectal bleeding, abdominal pain, palpable masses, weight loss, weakness, and fatigue [5]. Routine screening is vital for early detection and better outcomes in colorectal cancer management. While maintaining a healthy lifestyle for primary prevention can be challenging, regular screenings serve as a key strategy for secondary prevention, aiming to reduce mortality associated with the disease. Screenings are essential in combating colorectal cancer, given the difficulties of lifestyle modification for primary prevention [7]. Despite the clear advantages of screening, approximately one-third of adults aged 50–75 do not undergo recommended screening procedures [4].

### Limitations of Traditional CRC Screening Methods

Colorectal cancer (CRC) is the only cancer for which screening has been demonstrated to reduce cancer mortality in average-risk women and men. Various screening tests are available, each with its own strengths and limitations [7]. CRC screening guidelines differ by country, including the starting ages and screening strategies [8] and there are various screening techniques for colorectal cancer, some of which rely on stool analysis the guaiac-based fecal occult blood test (gFOBT) [9], fecal immunochemical test (FIT), while others involve direct visualization of the lesion the flexible sigmoidoscopy, the colonoscopy, computed tomography colonography (CTC) and colon capsule. Randomized controlled trials have consistently shown that both annual and biennial fecal occult blood tests (FOBTs) are associated with a significant reduction in colorectal cancer (CRC) mortality rates, with reductions ranging from 15% to 33% [6, 7]. The FIT (fecal immunochemical test) has higher sensitivity and specificity compared to that of gFOBT (guaiac-based fecal occult blood test) [5], first of all due to its ability to specifically target human globin, a protein component of the hemoglobin (Hb) molecule. FITs detect only human blood, using antibodies specific for human globin, unlike gFOBTs which can also detect other substances leading to false-positive results. This makes FITs less susceptible to interference from dietary factors and medications [10]. Additionally, FITs are more specific for bleeding from the lower gastrointestinal tract as globin is degraded by digestive enzymes in the upper gastrointestinal tract, enhancing their specificity for detecting neoplasia in the colon and rectum. Instead, gFOBT detects the presence of heme using an oxidation method and is therefore susceptible to interference resulting from either intake of antioxidants (increasing the risk of false negatives), or intake of heme in red meat or of peroxidase in fruits and vegetables (increasing the risk of false positives), resulting in lower sensitivity and specificity, respectively [11]. Furthermore, test is not specific to colonic bleeding, meaning detected heme can originate from the upper gastrointestinal tract [12]. According to a previous study, FIT has a sensitivity of 79% and a specificity of 94% [13]. The FIT is not influenced by the patient’s diet or medication taken in the preceding days, while the gFOBT is because the intake of NSAIDs or changes in diet can cause false positives, while the intake of vitamin C supplements can lead to false negatives [7]. However, there remains a risk of missing approximately half of early AA (advanced adenomas) and CRC (colorectal cancer) [14]. Flexible sigmoidoscopy presents itself as an alternative for directly examining the lower portion of the colon [15], but does not provide a comprehensive assessment of the entire colon. It’s limited to the distal portion, necessitating complementary screening modalities for a comprehensive evaluation, especially in high-risk populations. In populations with a higher risk, such as those with a family history of colon cancer, flexible sigmoidoscopy might not offer sufficient screening if the physician suspects that the patient’s problem is not confined to the lower part of the colon or rectum. However, some individuals may be unwilling or unable to undergo a full colonoscopy due to anxiety, claustrophobia, medical risks, or difficulties with bowel preparation, which is required for a colonoscopy compared to only an enema required for sigmoidoscopy [7]. Colonoscopy is considered a second-level examination for the diagnosis of colorectal cancer when a first-level screening test yields a positive result [15]. However, it’s important to consider the associated risks, as highlighted by a meta-analysis, which include a 4% risk of perforation and an 8% risk of bleeding per 10,000 colonoscopies [16]. Despite colonoscopy offering advantages in terms of higher sensitivity and specificity compared to most screening methods, it still has a 26% miss rate for adenomas [17], especially those located in the right colon. Additionally, it’s important to note limitations such as the requirement for thorough intestinal preparation by the patient and the need for sedation [3]. Computed tomography colonography (CTC) or virtual colonoscopy is a non-invasive imaging procedure that uses CT scans to produce detailed images of the colon. It offers advantages over traditional colonoscopy, such as being less invasive, having a lower risk of complications, and not requiring sedation. However, it requires bowel preparation, involves radiation exposure, and can detect incidental findings outside the intestine, possibly leading to additional tests and treatments. CTC usage is limited due to a lack of qualified radiologists and imaging centers. The colon capsule, a novel method, uses a wireless camera capsule to capture images of the colon mucosa without radiation, sedation, or gas insufflation, offering a groundbreaking approach to colorectal cancer screening [15]. Previous generations of the capsule had suboptimal accuracy [18]. In a screening study involving 884 individuals at average risk, of whom 695 (79%) were assessable, conventional adenomas of 6 mm or larger were identified with 88% sensitivity and 82% specificity. However, 26% of false-negative results were attributed to sessile serrated polyps [19].

### Potential of DNA Methylation Biomarkers for Early CRC Detection

The understanding of tumorigenesis in colorectal cancer (CRC) has evolved significantly, emphasizing the role of both genetic and epigenetic alterations in driving the disease process. Among these alterations, aberrant DNA methylation, particularly at CpG sites within gene promoters, has been recognized as a hallmark of cancer development, including CRC [20]. In CRC, the accumulation of genetic mutations and epigenetic changes, such as DNA methylation, leads to the dysregulation of key cellular pathways involved in cell growth, differentiation, and apoptosis. Aberrant methylation at specific CpG sites across the genome can silence tumor suppressor genes or activate oncogenes, contributing to the initiation and progression of CRC. One notable aspect of CRC detection is the potential utility of stool-based DNA testing [21]. Stool specimens from CRC patients often contain exfoliated tumor cells, providing a noninvasive source of tumor-derived DNA. By detecting methylated DNA of specific genes in stool samples, it’s possible to identify individuals with CRC or precancerous lesions, offering a promising avenue for early detection and intervention. Noninvasive stool-derived DNA testing holds considerable promise as a screening tool for CRC, as it offers advantages such as convenience, accessibility, and the potential for earlier detection compared to traditional screening methods like colonoscopy. By leveraging the molecular alterations associated with CRC, such as aberrant DNA methylation patterns, stool-based DNA testing can enhance the detection of CRC and improve patient outcomes through earlier diagnosis and intervention [5]. Currently, three non-invasive methylation biomarkers—N-Myc downstream-regulated gene 4 (NDRG4), Bone Morphogenetic Protein 3 (BMP3), and Septin 9 (SEPT9)—have received approval from the Food and Drug Administration (FDA) for CRC screening [5, 22]. Another test marker, VIM methylation, is currently commercially available but is awaiting FDA approval [23]. Stool samples exhibited notably higher positive detection rates for CRC (93.4%) and adenoma (81.3%) compared to normal samples, with a specificity of 94.3%. Furthermore, TFPI2 methylation in fecal samples from stage I to III CRC patients has shown promise as a biomarker for early CRC detection, with sensitivity ranging from 76% to 89% and specificity ranging from 79% to 93% [24–26]. Incorporating TFPI2 methylation into fecal DNA could enhance noninvasive CRC screening strategies. Generally, aberrantly methylated genes in solid tumors are suited biomarkers for early cancer detection and can be easily detected in stool samples. In a previous study, for example, the overall sensitivity of methylated SDC2 was 90.0% for CRC and 33.3% for advanced adenoma, with a specificity of 90.9% by Linear Target Enrichment-quantitative methylation-specific PCR in stool samples [27].

### Stool-Based DNA Methylation Tests

Economic development and the adoption of Western dietary habits in developing countries have led to a significant increase in the incidence and mortality of CRC. This trend underscores the importance of addressing lifestyle factors and implementing effective screening and prevention programs in these regions [21]. Distant metastasis in CRC is associated with a very low 5-year survival rate, often less than 10%. Early detection significantly improves the prognosis of CRC, with considerably higher survival rates when the cancer is diagnosed at an early stage. However, due to the lack of symptoms in the early stages and inadequate screening practices, many patients are diagnosed when the disease has already advanced. Given these challenges, the development of convenient and effective methods for the early diagnosis of CRC is crucial. Screening programs that target asymptomatic individuals or those at higher risk, coupled with advances in diagnostic technologies such as noninvasive stool-based DNA testing and imaging modalities, can play a significant role in improving early detection rates and ultimately reducing mortality from CRC. Additionally, raising awareness about the importance of regular screening and early detection among both healthcare providers and the general population is essential for combating CRC effectively [28]. Stool specimens from CRC patients often contain exfoliated tumor cells, which can release DNA into the stool. Detecting methylated DNA of specific genes associated with CRC in these noninvasive stool samples has emerged as a promising strategy for CRC detection and screening. This approach, known as stool DNA testing or fecal DNA testing, involves analyzing stool samples for the presence of aberrantly methylated DNA markers associated with CRC. These methylated DNA markers can serve as sensitive and specific biomarkers for the detection of CRC and precancerous lesions. By targeting specific genes known to be involved in CRC development and progression, stool DNA testing can identify individuals at increased risk of CRC who may benefit from further diagnostic evaluation, such as colonoscopy [29].

### Advantages of Stool-Based DNA Methylation Tests Over Traditional Methods

Stool-based screening for colorectal cancer (CRC) is promising due to its noninvasive nature and ease of sample collection. It relies on the detection of biomarkers from neoplastic cells shed into stool, offering a simple way to identify CRC or precancerous lesions. This approach is more acceptable to patients and may increase screening participation rates. The progression from benign polyps to malignant tumors in CRC involves genetic and epigenetic changes in colonic epithelial cells [30]. Stool-based screening methods can potentially detect these alterations, such as aberrant DNA methylation patterns or genetic mutations, providing valuable information for early detection and intervention [31]. However, there are still several challenges to address before fecal DNA-based tests can be utilized in clinical practice. One study compared a non-invasive, multitarget fecal DNA test to a fecal immunochemical test (FIT) in average-risk individuals for colorectal cancer [22]. The 9,989 participants were included in the study, with 65 having colorectal cancer and 757 presenting advanced precancerous lesions on colonoscopy. The DNA test exhibited a sensitivity of 92.3% (number 60) for detecting colorectal cancer and 42.4% (number 321) for advanced precancerous lesions (include advanced adenomas and sessile serrated polyps measuring 1 cm or more), surpassing FIT by an absolute difference of nearly 20 percentage points in both cases. So, the FIT had a sensitivity of 73.8% (number 44) for detecting colorectal cancer, while a sensitivity of 23.8% (number 180) for detecting precancerous lesions. The specificities with the DNA test and FIT were 86.6% and 94.9%, respectively, among participants with non-advanced or negative results, and 89.8% and 96.4%, respectively, among those with negative results on colonoscopy [22]. The characteristics of a screening test include its sensitivity and specificity. Sensitivity refers to the test’s ability to correctly identify individuals who have the disease, while specificity refers to the test’s ability to correctly identify individuals who do not have the disease. In summary, this study suggests that among asymptomatic individuals at average risk for colorectal cancer, the multitarget stool DNA test detected a significantly higher number of tumors compared to FIT. However, it also yielded more false-positive results, with lower specificity for the multitarget stool DNA test compared to FIT [22].

### Evaluation of Stool-Based DNA Methylation Tests

The interest in clinical applications has intensified with the emergence of epigenetic solutions for colorectal cancer (CRC) screening. These solutions rely on identifying specific epigenetic alterations in fecal DNA. Registered tests utilizing DNA methylation for CRC screening using fecal samples include Cologuard (Exact Science Co.), ColoClear (New Horizon Health Technology Company, Ltd.), Earlytect Colon (Genomictree, Inc.), Colosafe (Creative Biosciences Guangzhou Company), and Colowell (Shanghai Realbio Technology Company, Ltd.). Table 1. Cologuard and ColoClear analyze methylation of NDRG4 and BMP3, along with KRAS mutations, whereas the other tests rely on SDC2 methylation status. In 2014, Cologuard obtained full FDA approval for use in adults over 50 years old at average risk of CRC. In 2019, this indication was expanded to include younger individuals (aged 45 years and older). Combining an immunochemical assay for human hemoglobin with molecular genetic and epigenetic analyses, Cologuard shows significantly greater sensitivity in detecting CRC compared to the traditional fecal immunochemical test (FIT) (92.3% [95% confidence interval (CI), 83.0%–97.5%] vs. 73.8% [95% CI, 61.5%–84.0%]; *p* = .002). However, as per the trial (ClinicalTrials.gov identifier NCT01397747), FIT demonstrates higher specificity among participants with nonadvanced or negative findings (86.6% [95% CI, 85.9%–87.2%] vs. 94.9% [95% CI, 94.4%–95.3%]; *p* < .001) [22]. The NCCN Guidelines for CRC Screening (version 2.2022), the ACS CRC screening guideline (2018), and the USPSTF Screening for CRC recommendations (2021) all recommend the use of Cologuard as a CRC screening strategy. Both the NCCN and the ACS endorse the rescreening interval approved by the FDA, which is every 3 years. However, the USPSTF suggests testing every 1–3 years. EarlyTect-Colon Cancer (Genomictree, South Korea), approved by the Korean regulatory authority, and Colosafe (Creative Biosciences China), approved by the China National Medical Products Administration, are two stool-based DNA tests (sDNAs) [32, 33]. These two commercially available kits have been specifically designed to detect methylated Syndecan2 (SDC2) gene. Furthermore, iColocomf is designed to detect methylated SDC2 DNA along with TFPI2 in feces, thus expanding the options for colorectal cancer screening [33].

**TABLE 1:** Commercial methylation kits for colorectal cancer detection.

Product name	Target genes	Sample type	Sensitivity for CRC	Specificity for CRC	Related clinical trial (NCT ID) and related articles	Phase
Cologuard Exact Sciences Co. (USA)	NDRG4, BMP3 methylation+7 KRAS mutations	Stool DNA	92.3% (95% CI, 83.0–97.5)for detecting CRC	86.6% (95% CI, 85.9–87.2)	NCT01397747 NCT03741166 NCT03728348 [22]	FDA approved (2014)
Earlytect Colon Cancer Genomictree, Inc. (South Corea)	SDC2 methylation	Stool DNA	90.2% (95% CI, 85.8–93.6 n = 221/245) overall sensitivity 89.1% (95% CI, 82.3–93.9 n = 114/128) for detecting early stage (0-II)	90.2% (95% CI, 85.8–93.6 n = 221/245)	NCT03146520 NCT04304131 NCT05255588 [29, 32]	CE IVD (2017) MFDS approved (2018)
Colosafe Creative Biosciences (China)	SDC2 methylation	Stool DNA	84.22% (n = 315/374) in stage I-IV. 86.71% (n = 137/158) in stage I-II	97.85% (n = 821/839)	NCT04030637 NCT04786704 NCT05374369	NMPA approved (2018)
Coloclear New Horizon Health Tecchnology (China)	NDRG4, BMP3 methylation + KRAS mutations	Stool DNA	96% for CRC. 64% for advanced adenoma	87%	NCT042287335	NMPA approved (2020)
Colowell Shanghai Realbio Technology Company	SDC2 and SFRP2 methylation	Stool DNA			NCT04515082	
ColoSureTM	VIM methylation	Stool DNA	72%–77%	83%–94%	[12]	CLIA
iColocomf Wuhan Ammunition Life-tech Co.	SDC2 and TFPI2 methylation	Stool DNA	95.31%	90.3%	[33]	Trademark registered

## Discussion

Colorectal cancer represents the second most common neoplasm worldwide according to 2020 data of Global Cancer [1], with a high number of deaths. In order to achieve the greatest reduction in both incidence and mortality, the screening test should be capable of effectively identifying advanced precursor lesions as well as early-stage cancers across the entire colorectum. Additionally, it should be user-friendly, accessible, and affordable for patients. None of the conventional screening methods completely meet these desired criteria [34]. Due to its noninvasive nature and convenient benefits, along with colonoscopy, FIT is recommended for CRC screening in most guidelines [8]. The potential extent to which screening can prevent cases of CRC and associated deaths is likely substantial, although the exact magnitude remains uncertain. In principle, regular non-invasive monitoring could identify most early-stage or incipient CRCs, minimizing the risk of progression [7]. This is why innovative screening tests for colorectal cancer are currently being developed. An ideal screening test should exhibit high sensitivity and specificity, be cost-effective, easy to administer, and noninvasive or minimally invasive for patients.

In summary, fecal DNA methylation tests for CRC screening offer a promising approach due to their non-invasive nature, ease of administration, and potential for early detection. While they present several advantages, challenges such as false positives and false negatives remain. Clinical interest in epigenetic solutions for colorectal cancer (CRC) screening has grown, focusing on specific epigenetic alterations in fecal DNA. Tests like Cologuard, ColoClear, Earlytect Colon, Colosafe, and Colowell utilize DNA methylation for CRC screening. Further research is necessary to optimize their effectiveness and integrate them into clinical practice. The ultimate goal is to develop a simple, cost-effective, and accurate method for stratifying medium-risk populations and identifying individuals requiring further treatment for colorectal cancer or adenomas [8]. The exploration of DNA methylation patterns in colorectal cancer extends beyond tumor prevention, as methylated genes have also been studied for use in risk assessment, therapy monitoring, and prognosis prediction. These innovations offer new diagnostic and therapeutic avenues, significantly improving patient outcomes and marking a shift towards precision medicine in colorectal cancer care.
